# Endovascular recanalization of common carotid artery Total occlusion: two case reports and literature review

**DOI:** 10.1186/s42155-020-0097-6

**Published:** 2020-01-13

**Authors:** Jung-Chi Hsu, Han-Lin Tsai

**Affiliations:** 1 Department of Internal Medicine, Division of Cardiology, Saint Mary’s Hospital Luodong, Yilan, 26546 Taiwan; 2 Graduate Institute of Clinical Medicine, National Taiwan University College of Medicine, 7 Chung-Shan South Road, Taipei, 100 Taiwan; 3 Department of Internal Medicine, Ditmanson Medical Foundation Chiayi Christian Hospital, 539 Jhongsiao Road, Chiayi, 600 Taiwan

**Keywords:** Common carotid artery, Total occlusion, Endovascular therapy, Stroke

## Abstract

**Background:**

Common carotid artery total occlusion is rare but can be associated with a variety of neurological symptoms due to inadequate cerebral perfusion. The treatment includes bypass surgery, endarterectomy, and endovascular revascularization.

**Case presentation:**

Herein, we report two cases of common carotid artery total occlusion treated by percutaneous transluminal angioplasty, and review the literature.

**Conclusion:**

Both of our cases were successfully treated with endovascular revascularization for common carotid artery total stenosis. Endovascular therapy provided an alternative treatment. Further large clinical study for comparing the safety and efficiency in surgical and endovascular treatment may be required.

**Level of evidence:**

Level 4, Case Series.

## Background

Common carotid artery (CCA) total occlusion with concomitant ipsilateral internal carotid artery (ICA) stenosis is rare, with an incidence of less than 5% (Bajkó et al. 2013). The treatment is challenging. Chisci et al. reported two cases in which a hybrid technique was used for revascularization and showed good mid-term outcomes (Chisci et al. 2013). However, the safety of endovascular therapy is still controversial considering the possibility of distal embolization. With the use of protection devices, the endovascular approach has shown promise. Herein, we present two cases of CCA total occlusion that received endovascular revascularization.

## Case presentation

### Case 1

**Fig. 1:**
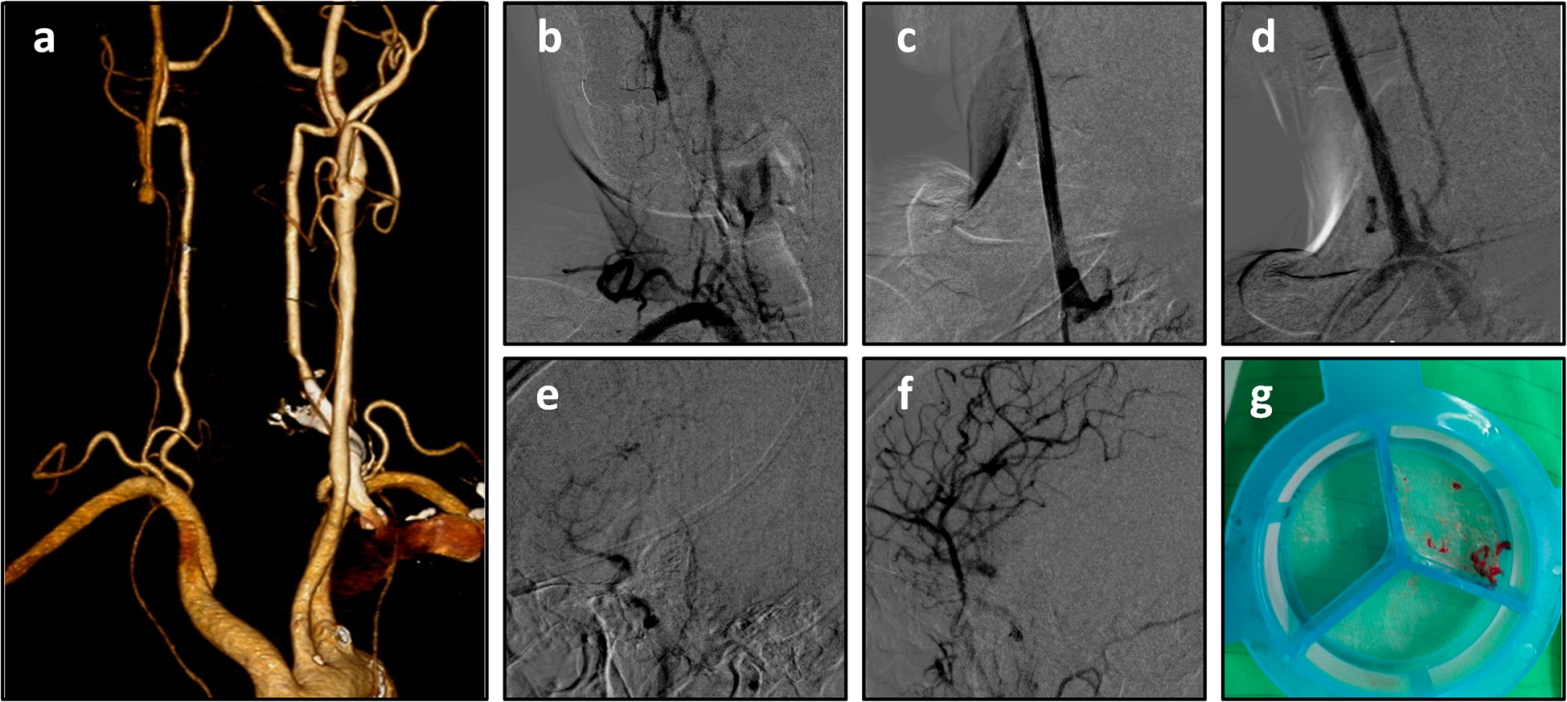
Patient 1 computed tomography angiography: right common carotid artery total occlusion (**a**). Angiography (**b**). Final result of angiography (**c**). Angiography 3 months later (**d**). Intracranial angiography before intervention (**e**). Intracranial angiography after the intervention (**f**). Thrombus (**g**)

A 71-year-old man with no underlying diseases presented to our neurology outpatient department due to dizziness and left upper extremity weakness for several days. Brain computed tomography (CT) showed unremarkable findings. Carotid duplex showed right CCA total occlusion with reversed right ophthalmic artery flow. CT angiography revealed right CCA long total occlusion (Fig. 1a), and angiography showed isolated common carotid artery occlusion (CCAO) (Fig. 1b, e).

A 6French 90-cm Flexor shuttle sheath (Cook medical, Bloomington, IN, USA) was inserted to the right CCA using a 0.035-in. guidewire (Terumo corporation, Japan) and a 5Fr JR 4 (Boston Scientific, USA) support. We tried to cross the CCA lesion with a Fielder Fc (ASAHI INTECC CO, Japan) wire using a Stride microcatheter (Asahi INTECC CO, Japan) but failed. The lesion was then crossed with a Conquest Pro wire (Asahi INTECC CO, Japan). The wire was advanced into the external carotid artery, and the Stride microcatheter was advanced to the distal CCA where it crossed the site of chronic total occlusion. The Fielder Fc wire was then advanced to the ICA, and the CCA lesion was dilated with a 2.0 mm/30 mm balloon to a maximum of 14 atm. A Filter wire crossed the CCA lesion, and a distal protection device (FilterWire EZ 190 cm, Boston Scientific, USA) was deployed in the ICA. Intravascular ultrasound (IVUS) was checked, and the lumen size was estimated. The CCA lesion was dilated with a 4/20 balloon to a maximum of 8 atm. A 7 Fr. Export aspiration catheter (Terumo, Japan) was used and several pieces of thrombi were retracted (Fig. 1g). 9.0 mm/50 mm and 9.0 mm/30 mm Wallstents (135 cm, Boston Scientific, USA) were deployed in the CCA, and the in-stent lesion was dilated with a 6.0/20 balloon to a maximum of 8 atm. There was good flow to the ICA and external carotid artery (ECA) after stenting (Fig. 1c, f). His symptoms improved after revascularization. Three months later, angiography showed a patent stent with complete expansion (Fig. 1d).

### Case 2

**Fig. 2:**
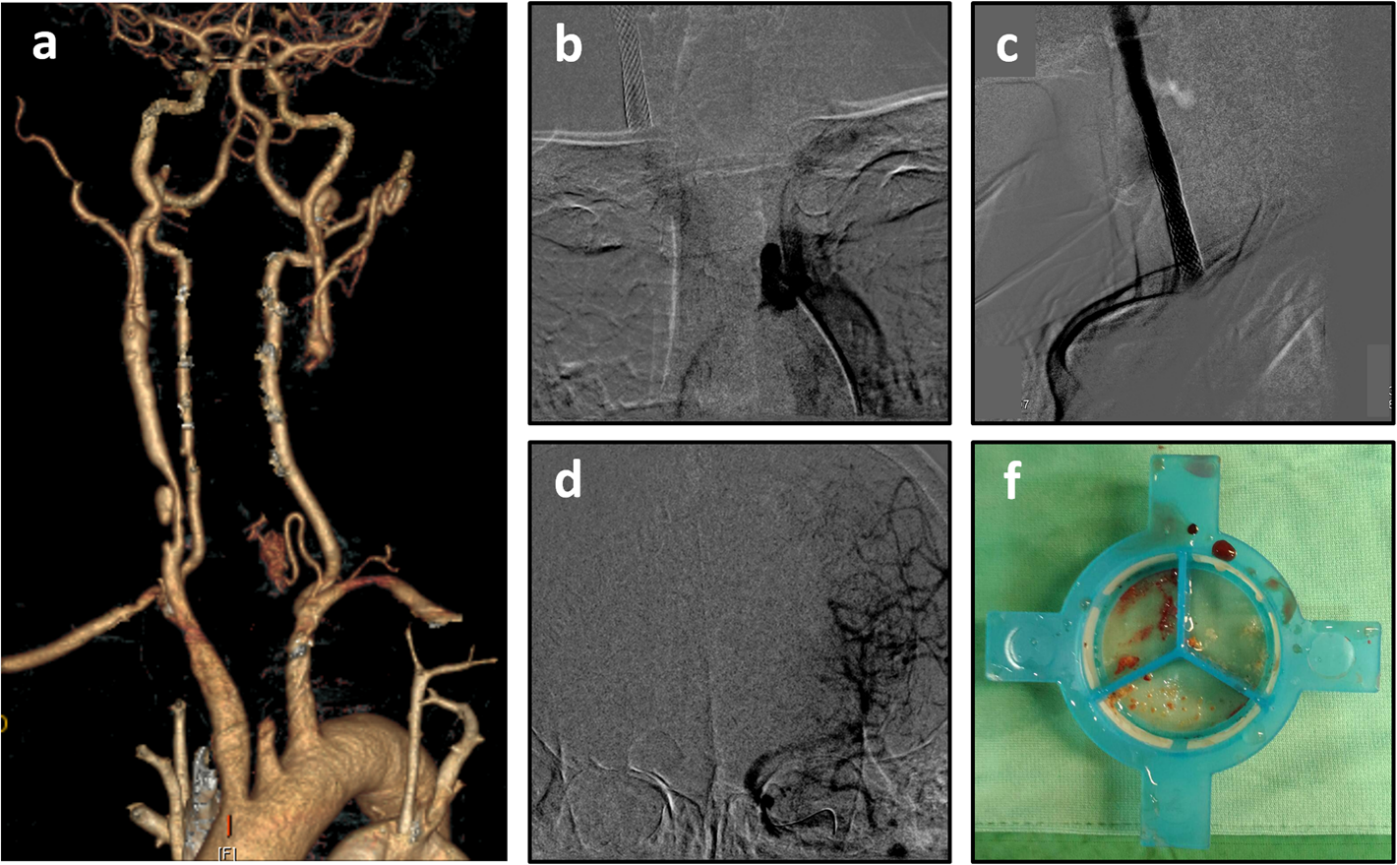
Patient 2 computed tomography angiography (**a**). Angiography (**b**). Final angiography (**c**). Intracranial angiography after the intervention (**d**). Thrombus (**e**)

A 53-year-old man presented with a medical history of nasopharyngeal carcinoma for which he had received radiotherapy 15 years previously. He complained of right upper limb weakness and difficulty in raising his hands for several months. In the recent 1 month, left upper limb weakness and dizziness were also noted. Carotid CT revealed right CCA dissection and left CCA total occlusion (Fig. 2a). Angiography showed 80% stenosis of the right CCA with a dissection flap. The left CCA was totally occluded, and flow arose from the left subclavian artery (Fig. 2b). A 9mmx40mm Wallstent (135 cm, Boston Scientific, USA) was deployed first in the right CCA. Six months later, he was admitted for left CCA stenting. An 8Fr JR4 was used to engage the CCA. We tried to cross the lesion with a Fielder Fc wire using a Strider microcatheter but failed. The wire was switched to a Conquest Pro wire, but this also failed. The CCA was then crossed with a Conquest 8/20 wire and dilated with a 1.2/6 balloon to 14 atm and a 2.0/20 balloon to 14 atm, and the filter wire crossed the CCA and was deployed in the ICA. A 7 Fr. Eliminate catheter was used, and some debris was retrieved (Fig. 2e). The CCA lesion was dilated with a 4/20 balloon to 14 atm. IVUS was performed, and we tried to deploy a 9/50 stent, but failed to cross the lesion. A 9/30 stent was deployed in the CCA to ICA. The in-stent lesion was dilated with a 4 mm/20 mm balloon to 14 atm and a 5 mm/15 mm balloon to 12 atm. Two stents were deployed in the CCA, and the in-stent lesion was dilated with a 6 mm/20 mm balloon to 10 atm. Blood flow in the CCA was subsequently regained (Fig. 2c, d). He was followed up at our outpatient department, and the symptoms of upper extremity weakness and dizziness both improved.

## Discussion and conclusions

Common carotid artery occlusion is rare and accounts for only about 0.24–5% of stroke patients. In 2013, Bajkó et al. analysed 5000 cases who received ultrasound and CT angiography, of whom 160 (3.2%) had carotid artery occlusion (Bajkó et al. 2013). Of these 160 patients, 20 (0.4%) presented with CCA occlusion or CCA occlusion associated with ICA occlusion. In another large ultrasonographic study of 6412 cases reported by Parthenis et al., the prevalence of CCAO was 0.54% (Parthenis et al. 2008). In addition, a case series by Chang et al. in 1995 showed that CAAO was almost equally distributed in both sides (51% located on the left) (Chang et al. 1995), and Belkin et al. in 1993 reported similar results (41% on the right side) (Belkin et al. 1993). A higher incidence was found in the patients who received surgical revascularization with patent distal vessels of left-sided occlusion (Parthenis et al. 2008). A possible explanation is that symptoms may be more obvious when left-sided occlusion occurs in right hemisphere-dominant people.

Various etiologies of CCAO have been reported. The major cause of CCAO is atherosclerosis, accounting for up to 75% of cases, and the strongest risk factors are hypertension and heart disease (Bajkó et al. 2013; Chang et al. 1995). Other causes include post-irradiation arteriopathy, cardiac embolism, dissection, aortic arch aneurysm, hypercoagulability, fibromuscular dysplasia, and Takayasu’s arteritis, which has been reported to be more common in Asian patients. One retrospective study reviewed 42 patients with Takayasu’s arteritis, and found that the CCA was the second most commonly involved vessel (47.6%), followed by the subclavian artery (50%) (Park et al. 2015).

The clinical manifestations of CCAO are associated with a variety of neurological symptoms, ranging from asymptomatic to severe cerebrovascular events. In addition, Man and Fu reported that patients who received neck irradiation for nasopharyngeal carcinoma only presented with isolated oculomotor nerve palsy (Man and Fu 2013). Since there are limited data in the literature and some patients do not have any symptoms, the true incidence of CCAO may be underestimated.

Collateral circulation can originate extracranially from the ipsilateral subclavian artery or intracranially from the circle of Willis, providing perfusion to the ipsilateral cerebral hemisphere in CCAO. Bajkó et al. reported an association between a higher incidence of occluded distal vessels and poorer short-term outcomes, however only two cases underwent revascularization surgery (Bajkó et al. 2013).

Two classification systems have been proposed. The first is classified by carotid duplex imaging, and depends on the patency and flow direction of the distal carotid vessels. Type I indicates isolated occlusion of the CCA and patent distal vessels, with flow directed from the external to ICA as type Ia and from the internal to external carotid artery as type Ib. Type II indicates isolated patent external carotid artery and type III indicated isolated patent ICA. Both vessels occlusion is classified as type IV. In this classification system, the flow direction in the ophthalmic and anterior or posterior communicating cerebral arteries is either antegrade or retrograde. Retrograde flow in the ophthalmic artery is more often associated with types II and IV lesions. Moreover, contralateral ICA stenosis has also been reported to be more severe in patients with type II and IV lesions (Parthenis et al. 2008).

The second classification system, known as Rile’s classification, is most widely used. According to the angiographic findings, type 1A involves a patent ICA and ECA, type 1B a patent ECA and an occluded ICA, type 1C a patent ICA and an occluded ECA, and type 2 total CCA, ICA, ECA occlusion (Klonaris et al. 2013). In our two patients, they were Rile’s type 1B occlusion.

The indication for CCAO intervention remains controversial, and the treatment has not been standardized. Surgical revascularization is possible in cases of patent distal vessels (Bajkó et al. 2013). A review study conducted by Klonaris in 2013 included 21 published studies and a total of 146 patients who underwent revascularization procedures for CCAO from 1965 to 2012 (Klonaris et al. 2013). The vast majority of the patients were treated with a surgical bypass procedure, and other techniques included transposition of the CCA to the subclavian artery, and endarterectomy (10%), whereas an endovascular procedure was performed in only one patient (0.9%) (Klonaris et al. 2013). However, this small percentage of using an endovascular approach may be based on the limited knowledge of this technique. First, chronic atherosclerotic plaques in CCA are usually heavy and calcified, which may restrict the guidewire from crossing the lesions, especially when they comprise the entire length of the CCA. Second, procedural risks such as embolic stroke is also a consideration. Finally, the prevalence and incidence of CCAO is rare, so that experience of this therapeutic approach is limited.

In our patients, the symptoms varied from dizziness to upper extremity weakness. During the procedure, a common characteristic was the presence of some thrombus or debris, indicating that the pathophysiology may not have been only atherosclerosis but also thrombosis, which is compatible with previous studies. The symptoms all improved after CCA revascularization. Experience of internal carotid stenting has shown the benefit of revascularization of chronic total occlusion (Chen et al. 2016); however, the field of endovascular interventions for CCA is limited. Although surgery is currently still the gold standard of treatment, endovascular treatment is an alternative choice that can provide adequate distal protection and thrombosuction.

Of note, we found thrombus and debris within the CCA in both of our cases. The longer the occlusion time, the more fragmented white debris and black blood clots were noted. From our experience, we suggest that routine IVUS and thrombosuction should be performed for CCA total occlusion. Common carotid artery total occlusion is rare and with variable etiology. The treatment is challenging with an endovascular approach. Both of our cases were successfully treated with endovascular revascularization for common carotid artery total stenosis. Endovascular therapy may be an alternative treatment other than surgery. Further large clinical study for comparing the safety and efficiency in surgical and endovascular treatment may be required.

## Data Availability

Not applicable.

## Abbreviations

CCA: Common carotid artery
CCAO: Common carotid artery occlusion
CT: Computed tomography
ECA: External carotid artery
ICA: Internal carotid artery
IVUS: Intravascular ultrasound

## References

[CR1] Bajkó Zoltán, Bălaşa Rodica, Moţăţăianu Anca, Maier Smaranda, Chebuţ Octavia Claudia, Szatmári Szabolcs (2013). Common Carotid Artery Occlusion: A Case Series. ISRN Neurology.

[CR2] Belkin M, Mackey WC, Pessin MS, Caplan LR, O'Donnell TF (1993). Common carotid artery occlusion with patent internal and external carotid arteries: diagnosis and surgical management. J Vasc Surg.

[CR3] Chang YJ, Lin SK, Ryu SJ, Wai YY (1995). Common carotid artery occlusion: evaluation with duplex sonography. AJNR Am J Neuroradiol.

[CR4] Chen YH, Leong WS, Lin MS, Huang CC, Hung CS, Li HY (2016). Predictors for successful endovascular intervention in chronic carotid artery total occlusion. JACC Cardiovasc Interv.

[CR5] Chisci E, Michelagnoli S, Frosini P, Ercolini L, Romano E, Setacci C (2013). An original technique for the treatment of symptomatic common carotid artery occlusion and concomitant ipsilateral internal carotid artery stenosis. J Cardiovasc Surg.

[CR6] Klonaris C, Kouvelos GN, Kafeza M, Koutsoumpelis A, Katsargyris A, Tsigris C (2013). Common carotid artery occlusion treatment: revealing a gap in the current guidelines. Eur J Vasc Endovasc Surg.

[CR7] Man BK, Fu YP (2013). Isolated oculomotor nerve palsy due to common carotid artery occlusion. BMJ Case Rep.

[CR8] Park BW, Park SJ, Park H, Hwang JC, Seo YW, Cho HR (2015). Stenosis or occlusion of the right subclavian and common carotid arteries is more common than that of the innominate artery in takayasu arteritis. Vasc Specialist Int.

[CR9] Parthenis DG, Kardoulas DG, Ioannou CV, Antoniadis PN, Kafetzakis A, Angelidou KI (2008). Total occlusion of the common carotid artery: a modified classification and its relation to clinical status. Ultrasound Med Biol.

